# Prenatal melamine, aromatic amine, and psychosocial stress exposures and their association with gestational diabetes mellitus in a San Francisco pregnancy cohort

**DOI:** 10.1038/s41370-025-00787-x

**Published:** 2025-06-30

**Authors:** Emily Lasher, Jessica Trowbridge, Alison Gemmill, Rachel Morello-Frosch, Erin DeMicco, Kurunthachalam Kannan, Jessie P. Buckley, Tracey J. Woodruff

**Affiliations:** 1 Department of Obstetrics, Gynecology and Reproductive Sciences, Program on Reproductive Health and the Environment, University of California, San Francisco, San Franscisco, USA CA; 2 Department of Population, Family, and Reproductive Health, Johns Hopkins Bloomberg School of Public Health, Baltimore, MD USA; 3 School of Public Health and Department of Environmental Science, Policy, and Management, University of California, Berkeley, CA USA; 4 Wadsworth Center, New York State Department of Health, Albany, NY USA; 5 Department of Epidemiology, Gillings School of Global Public Health, University of North Carolina at Chapel Hill, Chapel Hill, NC USA

**Keywords:** melamine, aromatic amines, o-anisidine, psychosocial stress, discrimination, gestational diabetes

## Abstract

**Background:**

Research suggests exposure to chemical and non-chemical stressors may increase the risk of pregnancy complications, including gestational diabetes mellitus (GDM). Exposure to melamine and aromatic amines (AAs) is ubiquitous among pregnant people. However, studies investigating the maternal and fetal health effects of prenatal exposure are limited.

**Objectives:**

This cross-sectional study aimed to (1) evaluate relationships between exposure to aromatic amines, melamine and its derivatives, and gestational diabetes in a pregnancy cohort in San Francisco, California, USA, (2) explore if non-chemical stressors modify these relationships, and (3) assess fetal sex differences using stratification.

**Methods:**

We measured 36 AAs, melamine, and three of its derivatives in second-trimester urine samples (*n* = 607). Financial strain and psychosocial stress were assessed using self-reported questionnaires. GDM status was abstracted from medical records. We used unadjusted and adjusted logistic regression models to calculate the odds of GDM associated with an interquartile range increase in urinary concentrations of melamine and AAs or higher levels of non-chemical stress, overall and stratified by infant sex. Interaction terms between each chemical and non-chemical stressor were used to test for effect modification.

**Results:**

Eight analytes were detected in >65% of participants, with 100% detection of melamine and cyanuric acid. Among male infants, summed urinary concentrations of melamine and its analogs and o-Anisidine were associated with increased odds of GDM (OR: 1.08 [1.00, 1.17], OR: 1.18 [1.03, 1.36], respectively). Higher levels of perceived stress and discrimination were also associated with increased odds of GDM (OR: 1.41 [0.73, 2.70], OR: 2.33 [1.16, 4.67], respectively). We found limited evidence of interaction between chemical and non-chemical stressors.

**Impact:**

This study revealed positive associations between melamine and its analogs, some aromatic amines, and gestational diabetes, especially among pregnant women carrying male fetuses. We also found that levels of perceived stress and discrimination were associated with gestational diabetes.

## Introduction

Tens of thousands of chemicals are in use in the United States, however, only a small fraction have been assessed for potential health effects [1]. Widespread use has resulted in ubiquitous human exposure to manufactured chemicals, including among pregnant people. However, very few have been assessed for impacts on pregnancy or fetal development. This is concerning because pregnancy is a vulnerable exposure window. Changes to vascular physiology, metabolism, reproductive organs, endocrine activity, and the immune system during pregnancy can heighten susceptibility to chemical and non-chemical stressors and associated maternal and fetal health risks [2, 3].

Melamine, an industrial chemical used in the production of plastics, adhesives, kitchenware, fertilizers, and laminates, is produced in high volumes in the United States (100,000,000 - <250,000,000 lb in 2019) [4]. Exposure to melamine stems from ingestion of contaminated food, water[5], and breast milk [6], indoor dust [7], and contact with clothing [8], and is frequently accompanied by exposure to structural analogs and degradation byproducts of melamine, such as cyanuric acid [9]. The International Agency for Research on Cancer (IARC) classified melamine as a possible human carcinogen [10]. Aromatic amines (AAs) are organic chemicals found in tobacco smoke, diesel exhaust, rubber, clothing and hair dyes, pesticides, food, and pharmaceuticals [11]. The IARC classified several AAs as “carcinogenic to humans” (i.e. o-toluidine, 2-naphthylamine, 4-aminobiphenyl) (Group 1), “probable human carcinogens” (i.e. aniline, o-anisidine) (Group 2a), and “possible human carcinogens” (i.e. 4-chloroaniline, 3,3-dimethylbenzidine) (Group 2b) [12]. Additionally, a number of AAs are included on California Proposition 65, a list of chemicals known to the state to cause cancer or reproductive toxicity [13].

A study from the National Institute of Health’s Environmental Influences on Child Health Outcomes (ECHO) program found detectable levels of melamine and its derivatives and several aromatic amines among 171 pregnant women across nine ECHO cohorts in the United States [14]. Melamine has also been detected in pregnant women in the Taiwan Maternal and Infant Cohort Study [15] and in the breast milk of women in Turkey [16] and the United States [6]. Further, a biomonitoring study in Brazil identified high urinary levels and detection rates of several AAs among 300 pregnant women [17]. Given evidence of widespread commercial use and emerging evidence demonstrating ubiquitous prenatal exposures, there is a need to investigate the potential maternal and fetal health effects of melamine and aromatic amine exposures.

Epidemiological studies on prenatal melamine exposure are limited, primarily exploring damage to the renal system [15], however, in animal studies, gestational exposure to melamine has been found to cause developmental toxicity [18, 19]. Melamine exposure has been associated with reduced male and female reproductive function in rodents [20–22]. Melamine may also disrupt the endocrine system [23], a property currently being evaluated by the European Chemicals Agency [24]. Gestational exposure to some AAs has been linked to embryonic [25] and reproductive toxicity [26, 27].

Past literature has demonstrated that exposure to endocrine-disrupting chemicals (EDCs) can increase the risk of gestational diabetes mellitus (GDM) [28, 29]. GDM is a high blood glucose condition that can develop during pregnancy [30] and is more common among pregnant people carrying a male fetus [31]. High levels of prenatal psychosocial stress have also been associated with GDM [32, 33], but it is unclear whether chemical and non-chemical stress exposures interact to amplify risk. Previous studies have illustrated the importance of evaluating joint effects, as exposure to cumulative stressors may amplify the risk of pregnancy complications [34]. Further, exposure to melamine and aromatic amines has been found to be higher among Hispanic and non-Hispanic Black pregnant women [14], increasing the likelihood of co-exposure to these stressors.

Thus, the main objectives of this study were to (1) evaluate relationships between exposure to aromatic amines, melamine and its derivatives, and gestational diabetes in a pregnancy cohort in San Francisco, California, USA (2), explore if psychosocial stressors and financial strain modify these relationships, and (3) assess fetal sex differences using stratification.

## Methods

### Data and study population

We analyzed data from participants of the Chemicals in Our Bodies (CIOB) cohort, a longitudinal pregnancy cohort in San Francisco, California. In 2016, CIOB joined the National Institute of Health’s Environmental Influences on Child Health Outcomes (ECHO) program. Participant recruitment methods have been reported previously [35–37]. Briefly, between 2014 and 2021, pregnant women were enrolled during their second trimester of pregnancy (between 12 and 28 weeks of gestation). To be eligible, women had to be over the age of 18, English or Spanish speaking, receiving prenatal care, and planning to deliver at San Francisco General Hospital or the University of California, San Francisco Moffit-Long/Mission Bay hospitals. Women with non-singleton pregnancies, diagnosed fetal anomalies, and diagnosed pregnancy complications were ineligible for participation. Participants provided written informed consent preceding study participation. The study protocol was approved by the Institutional Review Boards at the University of California, San Francisco (#13-12160) and the Johns Hopkins Bloomberg School of Public Health (#00027713).

Urine samples and questionnaire data were collected during the second trimester. Questionnaires assessed exposure characteristics and demographics, including race and ethnicity, maternal education, socioeconomic status, and marital status. Socioeconomic variables were used to calculate financial strain, a binary indicator considering the number of people in the household, household income, and the participant’s ability to pay for household basics. Questionnaires also measured participants’ perceived stress, depressive symptoms, and experiences of discrimination, which were used as indicators of psychosocial stress response, consistent with previous analyses [38–40]. Perceived stress was measured using the Perceived Stress Scale (PSS-4) [41]. Participants were asked a series of four questions about whether they experienced stressors in the past five years and were given a score between 0 and 16, with higher scores indicating higher levels of stress. For use in this analysis, scores were separated into tertiles, representing low (score 0–3), moderate (4-6), and high (7+) levels of perceived stress. Depressive symptoms were measured using the Center for Epidemiologic Studies Depression Scale (CES-D-10), a clinical screening tool that measures how often individuals experience depression symptoms in the past year in accordance with the Diagnostic Statistical Manual-IV [42]. Higher CES-D-10 scores suggest higher levels of depressive symptoms. CES-D-10 scores were also split into tertiles (0–4, 5–9, 10+). Additional information about the questions and scoring methods for these tools can be found in Tables S1–S2. Experiences of discrimination were assessed by asking, “How often do you feel that you, personally, have been discriminated against because of your race, ethnicity, ancestry, religion, or color?” Participants were asked to respond “never,” “rarely,” “sometimes,” “often,” or “very often.”

Health status indicators were obtained from medical records, including maternal age at delivery, pre-pregnancy body mass index, infant sex assigned at birth, and parity. GDM status (binary outcome; yes/no) was determined using clinical diagnoses that were abstracted from medical records. Participants with an unknown GDM status were excluded prior to analysis (*n* = 57).

### Analysis of urine samples

Urine samples were shipped on dry ice and analyzed at the Wadsworth Center Human Health Exposure Analysis Resource (HHEAR) laboratory. The urine samples were analyzed using high-performance liquid chromatography-tandem mass spectrometry for 36 AAs, melamine, cyanuric acid, ammelide, and ammeline, as described in detail elsewhere [14, 43, 44]. Quantification was based on isotope dilution using labeled internal standards. The limit of detection (LOD) ranged from 0.09 to 0.19 ng/ml for melamine and its derivatives, and that for AAs ranged from 0.03 to 0.2 ng/ml. Two samples were not assessed for AAs due to insufficient volume for analysis. Consequently, the sample size for melamine and its derivatives (*n* = 607) was slightly larger than for AAs (*n* = 605). A complete list of analyzed AAs can be found in Table S3. Several quality control (QC) samples were analyzed with every batch of samples. The HHEAR Urine QC Pools A and B were processed with each batch. For all analytes, the coefficients of variation were ≤20%, aside from melamine in one HHEAR QC Pool A (CV = 49), though the CV fell to an acceptable range (7.9) with the removal of an outlier.

### Statistical analysis

For melamine and its derivatives, to address chemical levels below the LOD, we utilized the instrument-reported values. For aromatic amine biomarker levels below the LOD, instrument-reported values were not available, so we imputed the value of the LOD divided by the square root of 2, a method utilized by the Centers for Disease Control and Prevention (CDC) [45].

Urinary biomarker concentrations are impacted by a participant’s hydration status; hence, we used specific gravity adjustment to account for differential dilution across urine samples. We utilized the Boeniger method, in which urinary biomarker concentrations are multiplied by the ratio of median specific gravity in the study sample to an individual’s specific gravity measure [46]. E_sg_, the specific gravity-adjusted exposure biomarker, can be defined as E_sg_ = E_O_ * (SG_median_ − 1)/(SG_O_ − 1), where E_o_ is the observed exposure biomarker concentration, SG_median_ is the median of specific gravity values in the study sample, and SG_o_ is the observed specific gravity value [46]. SG_O_ values of 1 were replaced with 1.0001 to prevent E_sg_ = ∞.

We described the demographic characteristics of the full and analytic samples and calculated the mean and standard deviation of the continuous variables and sample size (%) of the categorical variables. For each analyte, we calculated the detection frequency, geometric mean, geometric standard deviation, minimum and maximum, and 25th, 50th, and 75th percentiles of both the raw chemical values and the specific gravity-adjusted values. To assess combined exposure to multiple chemicals, we calculated the molar sum for each chemical class (∑Melamine and ∑AA) by dividing the chemical concentration by its molar weight and summing the ratios. Analytes with a detection frequency <20% were not included in the molar sums. We calculated Spearman correlation coefficients of specific gravity-adjusted chemical concentrations among analytes with ≥20% detection frequency.

We utilized unadjusted and adjusted logistic regression models to examine associations between chemical and non-chemical exposures and GDM. We evaluated whether associations differed by infant sex using stratification. Then we applied interaction terms to test if non-chemical stress levels modify the associations between chemical analytes and GDM. Measures of non-chemical stress included perceived stress, depressive symptoms, discrimination, and financial strain. For each combination of chemical and non-chemical stressors, we calculated the overall interaction *p*-value and the strata-specific odds ratios from the interaction model. Further, if *p* < 0.2, we visually verified evidence of interaction by plotting the regression with and without the interaction term.

Confounders adjusted for in the models were identified a priori and selected using a Directed Acyclic Graph (DAG), informed by existing literature (Fig. S1). Chemical models were adjusted for maternal age (continuous), race/ethnicity (White, Latinx, Asian, Other/Unknown), and education (some college or less, bachelor’s degree, graduate degree). Race/ethnicity was included as a proxy for structural racism, which is independently associated with chemical exposure, psychosocial stress, and GDM. Categories were collapsed for use in logistic regression models due to small numbers in some categories. Other/unknown includes Black, Native Hawaiian or Pacific Islander, Native American, multiracial, and individuals of unknown race. Education was included as an indicator of socioeconomic status. Models evaluating associations between psychosocial stress and GDM were adjusted for maternal age (continuous), race/ethnicity (White, Latinx, Asian, Other/Unknown), education (some college or less, bachelor’s degree, graduate degree), and parity (1+ births, no prior births). Interaction models were adjusted for maternal age (continuous), race/ethnicity (White, Latinx, Asian, Other/Unknown), and education (no graduate degree, graduate degree).

Logistic regression analyses were run on complete cases (chemical models: *n* = 605–607; stress models: *n* = 546–561) (Fig. S2). Participants were excluded from the analysis for missing GDM diagnosis (*n* = 57), maternal age (*n* = 5), education (*n* = 24), or specific gravity value (*n* = 7). An additional 42 participants were excluded from the stress models due to missing parity data. We modeled analytes with >65% of the sample above the LOD as continuous variables, as data visualization of Pearson residuals and locally weighted regression (loess) curves suggested the chemicals are linearly related to the log odds of GDM [47]. For each chemical, we calculated the interquartile range (IQR). Beta coefficients and 95% confidence intervals (CIs) were multiplied by the IQR and exponentiated to determine the odds ratio (OR) of GDM associated with each IQR increase in urinary concentration. We modeled analytes with a detection frequency between 20% and 65% as binary indicator terms (detected vs not detected). Analytes detected in less than 20% of participants were not included in the analysis. We presented the ORs in forest plots on a logarithmic scale. We used *α* = 0.05 for the main models and *α* = 0.2 for models evaluating effect modification. All analyses were conducted using Stata version 18.0.

## Results

### Participant health and demographics

Descriptive statistics were comparable across the full sample and analytic samples, including both the chemical models (Table 1) and the stress models (Table S4). In the chemical models, the majority of the participants were married or living with a partner (91%), had a bachelor’s (26%) or graduate degree (42%), identified as White (42%) or Latinx or Hispanic (30%), and did not experience financial strain (68%) (Table 1). The average age of participants at delivery was 33 (standard deviation (SD) = 5.0). Fifty-two percent of participants were having their first child. For measures of psychosocial stress, the mean PSS-4 score was 5 (SD = 2.7) out of a possible 16 points, and the mean CES-D-10 score was 7 (SD = 4.7). Approximately a quarter of participants self-reported that they sometimes, often, or very often experience discrimination. Levels of non-chemical stress varied by participant race/ethnicity (see Table S5). For the outcome, 90 participants had a GDM diagnosis (15%). Prevalence of GDM differed by race/ethnicity; 12.2% of participants identifying as White were diagnosed with GDM, while 15.8% of participants identifying as Latinx and 17.1% of participants identifying as Asian were diagnosed with GDM (Table S6).

**Table 1:** Characteristics of study participants between 2014 and 2021 in San Francisco, CA.

	Mean ± SD or *n* (%)	
	Full sample (*n* = 700)	Chemical models (*n* = 607)
*Covariates*		
Maternal age at delivery (years)	33 ± 5.2	33 ± 5.0
Race/ethnicity		
White	268 (38)	255 (42)
Latinx	245 (35)	184 (30)
Asian	118 (17)	111 (18)
Other or Unknown^a^	69 (10)	57 (9)
Education		
Some college or less	233 (35)	193 (32)
Bachelor’s degree	168 (25)	159 (26)
Graduate degree	259 (39)	255 (42)
Missing	40	0
Pre-pregnancy BMI (kg/m^2^)		
<18.5	15 (3)	14 (3)
18.5–<25	286 (54)	265 (56)
25–<30	130 (25)	115 (24)
≥30	95 (18)	79 (17)
Missing	174	134
Parity		
1+ births	324 (50)	273 (48)
No prior births	321 (50)	292 (52)
Missing	55	42
Infant sex assigned at birth		
Male	350 (51)	301 (50)
Female	341 (49)	304 (50)
Ambiguous or missing	9	2
Marital status		
Married/Living with Partner	566 (90)	527 (91)
Other	61 (10)	50 (9)
Missing	73	30
*Measures of Non-Chemical Stress*		
Financial strain		
Yes	236 (36)	187 (32)
No	422 (64)	406 (68)
Missing	42	14
Perceived stress (PSS-4)	5 ± 2.8	5 ± 2.7
Low (0–3)	186 (29)	174 (30)
Moderate (4–6)	268 (42)	244 (42)
High (7+)	191 (30)	160 (28)
Missing	55	29
Depressive symptoms (CES-D-10)	7 ± 4.8	7 ± 4.7
Low (0–4)	222 (34)	205 (35)
Moderate (5–9)	276 (42)	251 (43)
High (>10)	154 (24)	130 (22)
Missing	48	21
Discrimination^b^		
Never	225 (34)	207 (35)
Rarely	253 (39)	233 (40)
Sometimes/often/very often	178 (27)	148 (25)
Missing	44	19
GDM		
No	552 (86)	517 (85)
Yes	91 (14)	90 (15)
Missing	57	0

*SD* standard deviation, *GDM* gestational diabetes mellitus, *BMI* body mass index (calculated as kilograms per meter squared, categories are based on CDC guidelines), *PSS-4* Perceived Stress Scale, *CES-D-10* Center for Epidemiologic Studies Depression Scale.

^a^Race and ethnicity categories were collapsed for use in logistic regression models due to small numbers in some categories. In the analytical dataset (*n* = 607), other/unknown includes Black or African American (*n* = 26), Native Hawaiian or Pacific Islander (*n* = 5), Native American (*n* = 1), multiracial (*n* = 20), and individuals of unknown race (*n* = 5).

^b^Response to the question, “How often do you feel that you, personally, have been discriminated against because of your race, ethnicity, ancestry, religion, or color?”

### Distribution of chemical concentrations in urine

**Fig. 1:**
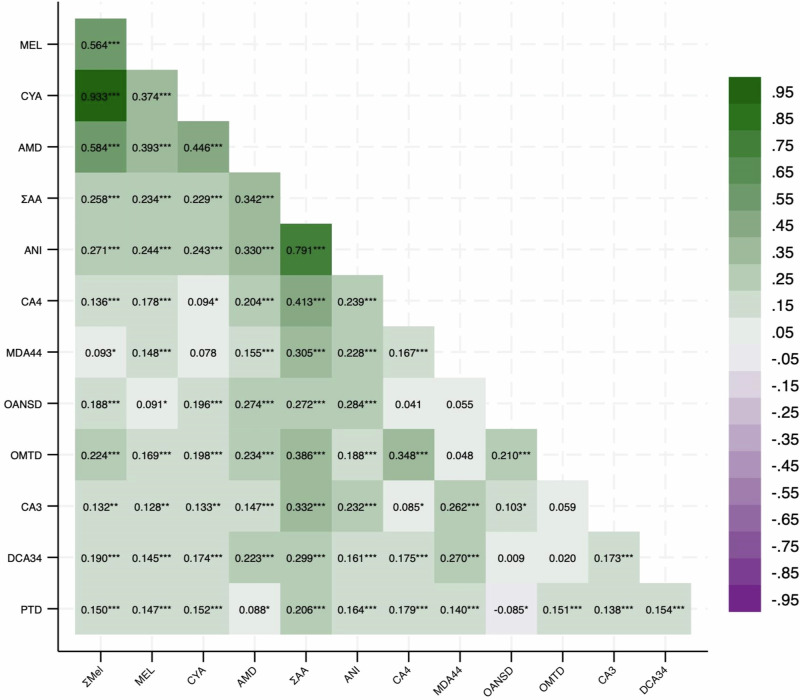
Spearman correlation coefficients of melamine, melamine derivatives, aromatic amine concentrations in urine, and their molar sums. *** *p* < 0.001, ** *p* < 0.01, * *p* < 0.05.

Melamine and cyanuric acid were detected in all participants, and ammelide was detected in 99% of urine samples at levels above the LOD (Table 2). Of the 36 aromatic amines, eight were detected in at least 20% of samples and utilized for analyses. The majority of aromatic amines tested in this cohort were excluded from our analysis due to low detection frequency. Among the chemicals, the highest geometric mean was observed for cyanuric acid (19.2 ng/mL), followed by aniline (12.2 ng/mL). The distribution of chemical concentrations only marginally differed when divided by participant race/ethnicity or level of stress (Table S7**;** Fig. S3). We detected low to moderate Spearman correlation coefficients between analytes (Fig. 1).

**Table 2:** Concentrations and distributions of urinary melamine and its analogs, and aromatic amines detected in ≥20% of samples from pregnant women in San Francisco (ng/mL).

Chemical			*n*	LOD	% Above LOD (*n*)^a^	*Unadjusted for SG*						*SG-adjusted*					
						GM (GSD)^b^	Min	Percentile			Max	GM (GSD)^b^	Min	Percentile			Max
								25	50	75				25	50	75	
	*Melamine & its analogs*																
Melamine		MEL	607	0.090	100 (607)	1.5 (3.0)	0.1	0.7	1.3	2.7	57	1.9 (3.3)	<LOD	0.9	1.6	3.3	4186
Cyanuric Acid		CYA	607	0.156	100 (607)	15.0 (2.5)	0.7	8.5	14.7	27.1	1160	19.2 (2.5)	0.7	11.4	17.1	26.4	16,240
Ammelide		AMD	607	0.135	99 (603)	3.1 (2.3)	<LOD	1.9	3.2	5.5	30	4.0 (2.6)	<LOD	2.4	3.9	6.2	325
ΣMelamine^c^		ΣMEL	607	NA	NA	NA	NA	NA	NA	NA	NA	0.2 (2.4)	<0.1	0.1	0.2	0.3	126
	*Aromatic amines*																
Aniline		ANI	605	0.100	96 (582)	9.5 (3.6)	<LOD	6.7	11	17.3	792	12.2 (4.0)	<LOD	7.5	12.2	21.2	102,760
4-Chloroaniline		CA4	605	0.050	85 (517)	0.6 (5.0)	<LOD	0.3	0.8	1.5	302	0.8 (4.6)	<LOD	0.4	0.8	1.7	174
4,4′-Methylenedianiline		MDA44	605	0.030	83 (505)	0.6 (5.7)	<LOD	0.4	1.0	1.8	43	0.8 (7.6)	<LOD	0.5	1.2	2.2	630
o-Anisidine		OANSD	605	0.030	69 (415)	0.3 (7.4)	<LOD	<LOD	0.4	1.4	50	0.4 (7.3)	<LOD	0.1	0.5	1.4	72
Composite of o/m-Toluidine		OMTD	605	0.050	88 (531)	1.1 (5.1)	<LOD	0.5	1.3	2.9	50	1.4 (4.4)	<LOD	0.8	1.4	3.0	252
3-Chloroaniline		CA3	605	0.050	40 (245)	NA	<LOD	<LOD	<LOD	0.8	252	NA	<LOD	<LOD	0.1	1.0	1778
3,4-Dichloroaniline		DCA34	605	0.100	31 (186)	NA	<LOD	<LOD	<LOD	2.3	50	NA	<LOD	<LOD	0.1	2.6	173
p-Toluidine		PTD	605	0.030	33 (201)	NA	<LOD	<LOD	<LOD	0.9	49	NA	<LOD	<LOD	0.04	1.1	29
ΣAA^c^		ΣAA	605	NA	NA	NA	NA	NA	NA	NA	NA	0.2 (3.2)	0	0.2	0.2	0.4	1106

*LOD* limit of detection, *GM* geometric mean, *GSD* geometric standard deviation, *Min* minimum, *Max* maximum, *SG* specific gravity

^a^Detection frequency was calculated from unadjusted values.

^b^Calculated for analytes with ≥65% above the LOD.

^c^Molar sums were calculated by summing the concentrations of frequently detected analytes (detection frequency >20%) scaled by their molecular weight. The units for these values are nmol/mL.

### Associations between chemical exposures, non-chemical stress, and GDM

#### Chemical stressors and GDM

**Fig. 2:**
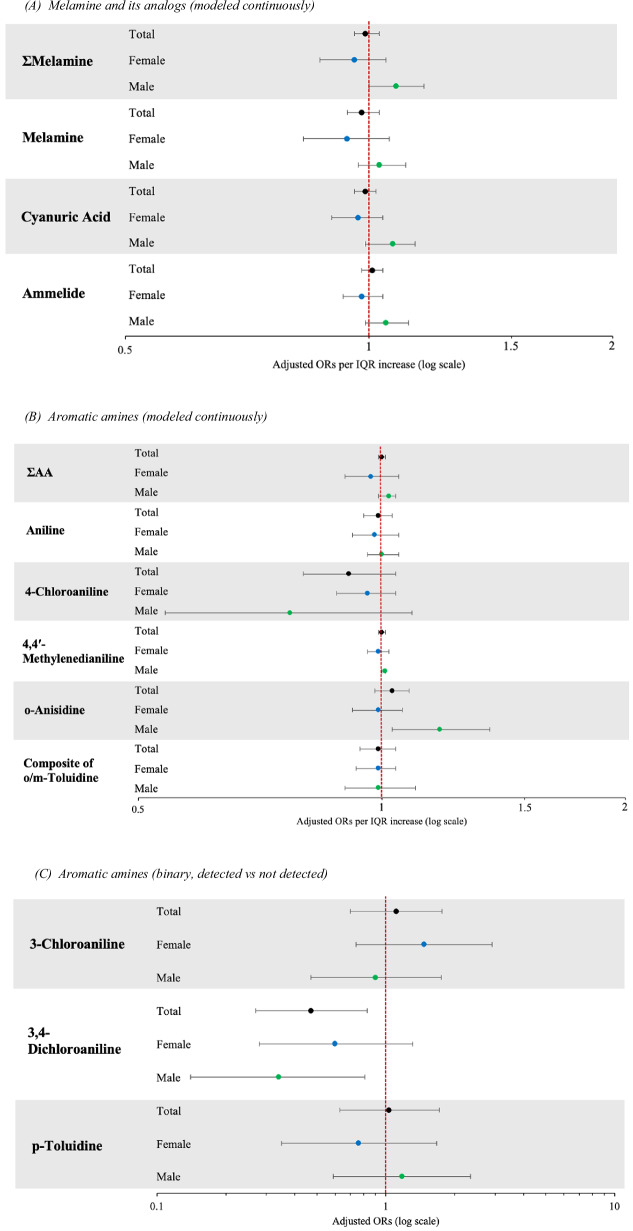
Adjusted odds ratios of the relationship between urinary chemical concentrations and gestational diabetes in total and sex-stratified models. Forest plots presenting the results of logistic regression models exploring the relationship between **A** melamine and its derivatives and GDM, chemical concentrations modeled continuously (*n* = 607, male=301, female=304); **B** aromatic amines and GDM, chemical concentrations modeled continuously (*n* = 605, male = 300, female = 303); and **C** aromatic amines and GDM, chemicals detected vs not detected (*n* = 605, male = 300, female = 303). Adjusted models control for maternal age, race/ethnicity, and education. Two participants with missing infant sex were excluded from sex-stratified models. The x-axis displays a logarithmic scale. OR odds ratio, IQR interquartile range.

Associations between melamine and its analogs and GDM were near-null in non-sex-stratified models. Melamine was weakly associated with higher odds of GDM among pregnant people with male infants (OR per IQR increase: 1.03 [0.97, 1.11]) but not female infants (OR per IQR increase: 0.94 [0.83, 1.06]), adjusting for maternal age, race/ethnicity, and education (Fig. 2A**;** Table S8). Urinary concentration of cyanuric acid was also weakly associated with higher odds of GDM among pregnant people with male infants (OR per IQR increase: 1.07 [0.99, 1.14]) but not female infants (OR per IQR increase: 0.97 [0.90, 1.04]). There were also sex differences in the relationship between ammelide and GDM, with higher odds of GDM among pregnant people with male infants (OR per IQR increase: 1.05 [0.99, 1.12]) but not female infants (OR per IQR increase: 0.98 [0.93, 1.04]). Combined urinary concentrations of melamine and its analogs (ΣMelamine) were associated with a modest 8% increase in the odds of GDM in pregnant people with male infants (OR per IQR increase: 1.08 [1.00, 1.17]), though this relationship was not observed among pregnant people with female infants (OR per IQR increase: 0.96 [0.87, 1.05]).

Models exploring relationships between aromatic amines and GDM generally found near-null associations, however, o-Anisidine was associated with increased odds of GDM among males (OR per IQR increase: 1.18 [1.03, 1.36]) (Fig. 2B**;** Table S8). Associations between the low-detect AAs and GDM showed imprecise confidence intervals (Fig. 2C**;** Table S8). Having levels of 3-Chloroaniline >LOD was associated with increased odds of GDM among participants carrying females (OR: 1.47 [0.74, 2.92]) but not males (OR: 0.90 [0.47, 1.75]), while having levels of p-Toluidine >LOD was associated with increased odds of GDM among participants carrying males (OR: 1.18 [0.59, 2.35]) but not females (OR: 0.76 [0.35, 1.67]). Having levels of 3,4-Dichloroaniline >LOD was associated with lower odds of GDM (OR: 0.47 [0.27, 0.83]).

#### Non-chemical stressors and GDM

**Fig. 3:**
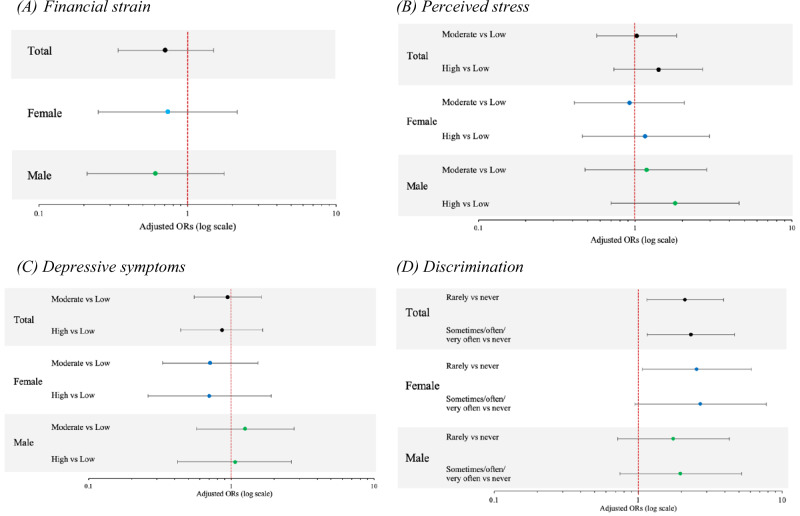
Adjusted odds ratios of the relationship between non-chemical stressors and gestational diabetes in total and sex-stratified models. Forest plots presenting the results of logistic regression models exploring the relationship between **A** financial strain and GDM (*n* = 561, male = 281, female = 279), **B** perceived stress (PSS-4) and GDM (*n* = 546, male = 270, female = 275); **C** depressive symptoms (CES-D-10) and GDM (*n* = 555, male = 278, female=276); **D** discrimination and GDM (*n* = 553, male = 274, female = 278). Adjusted models control for maternal age, race/ethnicity, education, and parity. One participant with missing infant sex was excluded from sex-stratified models. The x-axis displays a logarithmic scale. OR = odds ratio.

Financial strain had an OR < 1 for GDM (Fig. 3A**;** Table S9). Higher levels of perceived stress and discrimination were associated with increased odds of GDM. Compared to those with lower levels of perceived stress (PSS-4 score of 0–3), participants with a PSS-4 score of 7 or higher had a 41% increase in the odds of GDM, controlling for parity, maternal age, education, and race/ethnicity (OR: 1.41 [0.73, 2.70]) (Fig. 3B, Table S9). Participants who stated that they experienced discrimination sometimes, often, or very often had significantly increased odds of GDM compared to those who never experienced discrimination (OR: 2.33 [1.16, 4.67]) (Fig. 3D**;** Table S9).

Depression (CES-D-10 score >10) showed sex differences, with higher odds of GDM in males (OR: 1.06 [0.42, 2.63]) and lower odds in females (OR: 0.70 [0.26, 1.90]), though the confidence intervals were imprecise (Fig. 3C**;** Table S9). Limiting the models to the same analytic population had minor impacts on the measures of association and associated confidence intervals (Table S9).

#### Joint effects of chemical and non-chemical stress

We observed minimal interaction between chemical and non-chemical stressors (Table 3). We found evidence that discrimination and aniline may interact to increase risk of GDM [interaction term *p*-value = 0.2, odds of GDM per IQR increase in aniline among those who never experience discrimination = 1.00 (0.96, 1.04), sometimes or often or very often experience discrimination = 1.33 (0.95, 1.87)]. Associations between p-Toluidine (detected vs. not detected) and GDM were also stronger among those experiencing greater levels of discrimination [interaction term *p*-value = 0.0536, odds of GDM among those who never experience discrimination = 1.33 (0.51, 3.44), sometimes or often or very often experience discrimination = 2.32 (0.95, 5.69)]. The effect of o-Anisidine on GDM may have also been modified by discrimination level. The interaction term was not statistically significant (*p*-value = 0.2299), though the strata-specific ORs did show a similar trend: odds of GDM per IQR increase in o-Anisidine among those who never experience discrimination = 1.00 (0.90, 1.12), sometimes or often or very often experience discrimination = 1.30 (0.98, 1.71).

**Table 3:** Interaction between chemical and non-chemical stressors on the odds of gestational diabetes in logistic regression models.

Chemical	Measure of non-chemical stress		Strata-specific ORs *(interaction model)*	Overall *p*-value *(interaction term)*
ΣMelamine	Financial Strain	No	0.99 (0.97–1.02)	0.6633
		Yes	0.93 (0.67–1.28)	
	Perceived Stress Scale (PSS-4)	Low (0–3)	1.00 (0.97–1.02)	0.6826
		Moderate (4-6)	0.98 (0.89–1.08)	
		High (7+)	0.87 (0.62–1.21)	
	Depressive Symptoms (CES-D-10)	Low (0–4)	0.99 (0.94–1.05)	0.9397
		Moderate (5–9)	1.00 (0.95–1.05)	
		High ( > 10)	0.95 (0.72–1.26)	
	Discrimination	Never	1.02 (0.97–1.07)	0.5626
		Rarely	0.91 (0.74–1.13)	
		Sometimes/often/very often	1.00 (0.96–1.03)	
Melamine	Financial Strain	No	0.99 (0.95–1.04)	0.3370
		Yes	0.84 (0.60–1.17)	
	Perceived Stress Scale (PSS-4)	Low (0–3)	1.00 (0.95–1.04)	0.6110
		Moderate (4-6)	0.98 (0.90–1.07)	
		High (7 + )	0.87 (0.67–1.14)	
	Depressive Symptoms (CES-D-10)	Low (0–4)	0.98 (0.90–1.07)	0.9606
		Moderate (5–9)	0.99 (0.93–1.05)	
		High ( > 10)	0.96 (0.76–1.20)	
	Discrimination	Never	1.01 (0.96–1.07)	0.3853
		Rarely	0.84 (0.65–1.09)	
		Sometimes/often/very often	1.00 (0.99–1.01)	
Cyanuric Acid	Financial Strain	No	0.99 (0.97–1.02)	0.7051
		Yes	0.95 (0.74–1.21)	
	Perceived Stress Scale (PSS-4)	Low (0–3)	1.00 (0.97–1.02)	0.7012
		Moderate (4–6)	0.98 (0.90–1.07)	
		High (7 + )	0.92 (0.75–1.13)	
	Depressive Symptoms c(CES-D-10)	Low (0–4)	0.99 (0.95–1.04)	0.9090
		Moderate (5–9)	1.00 (0.96–1.04)	
		High ( > 10)	0.95 (0.76–1.18)	
	Discrimination	Never	1.01 (0.97–1.06)	0.6390
		Rarely	0.94 (0.80–1.10)	
		Sometimes/often/very often	1.00 (0.96–1.03)	
Ammelide	Financial Strain	No	1.01 (0.98–1.04)	0.8602
		Yes	0.99 (0.85–1.16)	
	Perceived Stress Scale (PSS-4)	Low (0–3)	1.02 (0.99–1.05)	0.6814
		Moderate (4–6)	1.00 (0.95–1.05)	
		High (7 + )	0.90 (0.64–1.27)	
	Depressive Symptoms (CES-D-10)	Low (0–4)	1.00 (0.94–1.05)	0.6594
		Moderate (5–9)	1.01 (0.98–1.05)	
		High ( > 10)	1.11 (0.87–1.40)	
	Discrimination	Never	1.02 (0.99–1.05)	0.8159
		Rarely	0.98 (0.89–1.08)	
		Sometimes/often/very often	1.01 (0.93–1.10)	
ΣAA	Financial Strain	No	1.00 (0.99–1.00)	0.3886
		Yes	0.86 (0.61–1.21)	
	Perceived Stress Scale (PSS-4)	Low (0–3)	1.01 (0.99–1.04)	0.5415
		Moderate (4–6)	0.97 (0.88–1.08)	
		High (7 + )	0.90 (0.65–1.22)	
	Depressive Symptoms (CES-D-10)	Low (0–4)	0.85 (0.64–1.14)	0.5676
		Moderate (5–9)	1.00 (1.00–1.01)	
		High ( > 10)	0.99 (0.82–1.20)	
	Discrimination	Never	1.00 (1.00–1.01)	0.6221
		Rarely	0.97 (0.88–1.06)	
		Sometimes/often/very often	1.09 (0.84–1.40)	
Aniline	Financial Strain	No	1.00 (0.97–1.03)	0.1398*
		Yes	0.65 (0.37–1.14)	
	Perceived Stress Scale (PSS-4)	Low (0–3)	1.00 (0.95–1.04)	0.8214
		Moderate (4–6)	0.99 (0.92–1.07)	
		High (7 + )	0.87 (0.57–1.33)	
	Depressive Symptoms (CES-D-10)	Low (0–4)	0.87 (0.64–1.17)	0.6283
		Moderate (5–9)	1.00 (0.98–1.01)	
		High ( > 10)	1.02 (0.84–1.24)	
	Discrimination	Never	1.00 (0.96, 1.04)	0.2000*
		Rarely	0.97 (0.88, 1.06)	
		Sometimes/often/very often	1.33 (0.95, 1.87)	
4-Chloroaniline	Financial Strain	No	0.93 (0.82–1.06)	0.6172
		Yes	0.87 (0.67–1.12)	
	Perceived Stress Scale (PSS-4)	Low (0–3)	0.79 (0.49–1.28)	0.8170
		Moderate (4–6)	0.92 (0.76–1.11)	
		High (7 + )	0.93 (0.80–1.07)	
	Depressive Symptoms (CES-D-10)	Low (0–4)	0.84 (0.57–1.24)	0.2830
		Moderate (5–9)	0.97 (0.90–1.06)	
		High ( > 10)	0.60 (0.31–1.16)	
	Discrimination	Never	0.87 (0.62–1.22)	0.6881
		Rarely	0.96 (0.86–1.08)	
		Sometimes/often/very often	0.82 (0.53–1.26)	
4,4′-Methylenedianiline	Financial Strain	No	1.00 (0.99–1.01)	0.5080
		Yes	0.96 (0.86–1.08)	
	Perceived Stress Scale (PSS-4)	Low (0–3)	1.01 (0.99–1.03)	0.4426
		Moderate (4–6)	0.99 (0.95–1.03)	
		High (7 + )	0.97 (0.88–1.08)	
	Depressive Symptoms (CES-D-10)	Low (0–4)	0.93 (0.76–1.16)	0.5779
		Moderate (5–9)	1.00 (1.00–1.01)	
		High ( > 10)	0.91 (0.71–1.15)	
	Discrimination	Never	1.00 (1.00–1.01)	0.6585
		Rarely	1.02 (0.94–1.10)	
		Sometimes/often/very often	0.92 (0.76–1.12)	
o-Anisidine	Financial Strain	No	1.01 (0.95–1.08)	0.2896
		Yes	1.06 (0.99–1.15)	
	Perceived Stress Scale (PSS-4)	Low (0–3)	1.10 (0.99–1.22)	0.2333
		Moderate (4–6)	0.93 (0.77–1.12)	
		High (7 + )	1.01 (0.95–1.09)	
	Depressive Symptoms (CES-D-10)	Low (0–4)	1.11 (0.98–1.26)	0.4595
		Moderate (5–9)	1.01 (0.94–1.09)	
		High ( > 10)	1.03 (0.96–1.09)	
	Discrimination	Never	1.00 (0.90–1.12)	0.2299
		Rarely	1.02 (0.96–1.08)	
		Sometimes/often/very often	1.30 (0.98–1.71)	
Composite of o/m-Toluidine	Financial Strain	No	0.98 (0.93–1.04)	0.6635
		Yes	1.01 (0.91–1.12)	
	Perceived Stress Scale (PSS-4)	Low (0–3)	1.02 (0.95–1.09)	0.6383
		Moderate (4–6)	0.95 (0.84–1.09)	
		High (7 + )	0.96 (0.81–1.15)	
	Depressive Symptoms (CES-D-10)	Low (0–4)	1.00 (0.94–1.08)	0.8760
		Moderate (5–9)	0.97 (0.88–1.07)	
		High ( > 10)	0.98 (0.89–1.09)	
	Discrimination	Never	1.01 (0.95–1.08)	0.6621
		Rarely	0.96 (0.85–1.07)	
		Sometimes/often/very often	1.02 (0.81–1.29)	
3-Chloroaniline	Financial Strain	No	1.52 (0.87–2.66)	0.0610*
		Yes	0.57 (0.24–1.35)	
	Perceived Stress Scale (PSS-4)	Low (0–3)	1.57 (0.64–3.85)	0.4893
		Moderate (4–6)	1.25 (0.60–2.63)	
		High (7 + )	0.75 (0.32–1.78)	
	Depressive Symptoms (CES-D-10)	Low (0–4)	0.92 (0.41–2.05)	0.7983
		Moderate (5–9)	1.33 (0.65–2.72)	
		High ( > 10)	1.16 (0.44–3.01)	
	Discrimination	Never	1.65 (0.65–4.22)	0.6275
		Rarely	1.08 (0.53–2.20)	
		Sometimes/often/very often	0.90 (0.39–2.08)	
3,4-Dichloroaniline	Financial Strain	No	0.49 (0.24–0.99)	0.9328
		Yes	0.46 (0.18–1.22)	
	Perceived Stress Scale (PSS-4)	Low (0–3)	0.39 (0.12–1.21)	0.3224
		Moderate (4–6)	0.79 (0.33–1.88)	
		High (7 + )	0.29 (0.10–0.83)	
	Depressive Symptoms (CES-D-10)	Low (0–4)	0.29 (0.09–0.88)	0.2801
		Moderate (5–9)	0.78 (0.36–1.73)	
		High ( > 10)	0.33 (0.09–1.23)	
	Discrimination	Never	0.78 (0.27–2.27)	0.1552*
		Rarely	0.60 (0.27–1.33)	
		Sometimes/often/very often	0.14 (0.03–0.61)	
p-Toluidine	Financial Strain	No	0.95 (0.50–1.80)	0.8876
		Yes	1.03 (0.45–2.32)	
	Perceived Stress Scale (PSS-4)	Low (0–3)	1.16 (0.45–3.01)	0.4802
		Moderate (4–6)	0.71 (0.30–1.67)	
		High (7 + )	1.47 (0.63–3.40)	
	Depressive Symptoms (CES-D-10)	Low (0–4)	0.61 (0.25–1.48)	0.3764
		Moderate (5–9)	1.38 (0.64–2.96)	
		High ( > 10)	1.05 (0.40–2.74)	
	Discrimination	Never	1.33 (0.51–3.44)	0.0536*
		Rarely	0.52 (0.22–1.22)	
		Sometimes/often/very often	2.32 (0.95–5.69)	

All models control for maternal age, race/ethnicity, and education.

*OR* odds ratio, *CES-D-10* Center for Epidemiologic Studies Depression Scale.

*p < 0.2.

However, interaction terms between financial strain and aniline, financial strain and 3-Chloroaniline, and discrimination and 3,4-Dichloroaniline demonstrated that higher levels of non-chemical stress may *decrease* the strength of the chemical association. For instance, we found evidence that the association between aniline and GDM may be weaker among those experiencing financial strain [interaction term *p*-value = 0.1398, odds of GDM per IQR increase in aniline among those who experience financial strain = 0.65 (0.37–1.14) vs. those who do not = 1.00 (0.97–1.03)].

## Discussion

In this San Francisco-based pregnancy cohort, exposure to melamine and its analogs, and several aromatic amines was ubiquitous among participants. Higher summed concentrations of melamine and its analogs were associated with small increases in the odds of GDM among pregnant people with infants assigned male at birth. Higher urinary concentrations of o-Anisidine, an aromatic amine, were also significantly associated with increased odds of GDM among males. This study contributes to the literature on the modifiable risk factors for GDM.

The biological mechanisms by which environmental exposures induce the development of GDM may include insulin resistance, β-cell dysfunction, neurohormonal dysfunction, inflammation, oxidative stress, epigenetic modification, and alterations in the gut microbiome [28]. Given its possible endocrine-disrupting properties [23], melamine may increase the risk of GDM by damaging pancreatic β-cells, which are then unable to balance increased insulin demand during pregnancy, similar to other endocrine disruptors such as polychlorinated biphenyls, phthalates, and per- and poly-fluoroalkyl substances [29]. Toxicological studies have also found evidence that melamine exposure may induce kidney injury via increased oxidative stress [48] and inflammation [49]. Exposure to melamine and cyanuric acid has also been shown to increase oxidative stress in the central nervous system [50]. Excessive oxidative stress has been associated with impaired lipid and glucose metabolism and increased expression of proinflammatory cytokines, which can aggravate insulin resistance, leading to hyperglycemia [28].

Literature on the potential underlying mechanisms between aromatic amine exposure and GDM is limited. One study of Brazilian pregnant women found that prenatal exposure to aromatic amines could induce oxidative stress with DNA damage, as women showed elevated levels of urinary 8OHdG, a biomarker for oxidative stress and carcinogenesis [17]. Another study suggested that 3,4-DCA may disrupt steroid hormone levels and steroidogenesis in zebrafish, which could influence the development of GDM [51]. Interestingly, our findings showed *lower* odds of GDM with 3,4-DCA, which contradicts this proposed mechanism. Future studies should further investigate how 3,4-DCA and other aromatic amines may contribute to hormone imbalances that affect GDM risk. Additional research on the reproductive and metabolic toxicity of o-Anisidine is also needed, as there is limited literature on non-cancer endpoints [12].

For most chemical and non-chemical stressors, we observed higher odds of GDM among pregnant people carrying infants assigned male at birth. This finding is supported by previous work on this topic; one systematic review and meta-analysis of 20 primary studies reported that pregnant women carrying a male fetus had a 4% higher relative risk of GDM compared to women carrying a female fetus (RR 1.04; 95% CI 1.02, 1.06), likely due to poorer maternal beta cell function [31].

We did not find evidence of an association between financial strain and GDM in this cohort. Previous literature on the relationship between financial status (e.g., income, poverty) and GDM is conflicting [52, 53]. However, the current study provides evidence that exposure to non-chemical psychosocial stressors may increase the risk of GDM. Psychosocial stressors such as life adversity, chronic work stress, and depression have been associated with glucose abnormalities in the general non-pregnant population [54, 55]. Recent studies have identified high levels of psychosocial stress during pregnancy as a potential risk factor for GDM. It has been hypothesized that psychosocial stress leads to hyperactivity of the hypothalamic–pituitary–adrenal (HPA) axis, increasing the production of cortisol, a stress hormone, affecting the pancreatic cells, and leading to insulin resistance [32]. Cortisol and other inflammatory markers are already elevated during pregnancy, which is believed to enhance insulin resistance that leads to the development of GDM [56]. Alternatively, psychosocial stress may also lead to physical inactivity, unhealthy diet, and obesity, which could increase blood lipid and glucose levels [57].

For perceived stress, though the confidence intervals were imprecise in our cohort, we did observe increased odds of GDM associated with higher PSS-4 scores, consistent with previous epidemiologic studies. In 2020, a prospective case-control study in India found that the odds of GDM were 13 times higher among those with high prenatal perceived stress compared to those with low prenatal perceived stress [32]. Similarly, a prospective cohort study of 1,115 Hispanic women in Massachusetts found that for every one-point increase in perceived stress score, participants had a 5.5 mg/dL increase in screening glucose level (*β* = 5.5; standard deviation = 2.8; *p* = 0.05) [58].

The current study did not find clear evidence of an association between depressive symptoms and GDM. Research has hypothesized that the relationship between depression and GDM is bidirectional, meaning that depression during pregnancy may increase risk of GDM, and the diagnosis of GDM may lead to an increased risk of depression during pregnancy and in the postpartum period [59]. Literature on the former is limited and conflicting [60]. Several studies have found no association between depression in early pregnancy and GDM [56, 61]. Other studies have found a significant association. For example, a 2022 retrospective cohort study by Thiele and colleagues observed increased odds of GDM for patients with antenatal depression or anxiety [adjusted OR: 1.15 (95% CI: 1.1, 1.19)] [62]. Risk was highest for individuals with no pre-pregnancy mental health history [62]. One recent systematic review found that depression was significantly associated with an increased risk of GDM [OR: 1.29 (95% CI: 1.10, 1.52)], a conclusion of which was derived from a limited number of studies (*n* = 9) with significant heterogeneity in the measurement of depression (i.e. timing, diagnostic criteria) [63].

Though racial discrimination is an important source of stress for racial and ethnic minorities and has been found to increase risk of adverse pregnancy outcomes (e.g., preterm birth, small for gestational age) [64], few studies have explored the association between experiences of discrimination and GDM. This analysis contributes to the limited literature on this topic, providing evidence that experience of discrimination increases risk of GDM. One prospective cohort study (*n* = 744) in the US between 2013 and 2015 found that exposure to discrimination was associated with increased odds of developing GDM [OR: 2.11 (95% CI: 1.03, 4.22)] [65]. Interestingly, the authors also found that more than 20% of the association operates via a pathway that includes obesity [65]. A cross-sectional study using 2012–2014 data from the New York City Pregnancy Risk and Assessment Monitoring System survey (*n* = 4084) observed that racial discrimination was associated with a 24% increase risk of GDM [adjusted RR: 1.24 (95% CI: 0.87, 1.78)] [40]. Several studies on non-pregnant populations have also found relationships between racial discrimination and insulin resistance [66, 67] and glucose intolerance [68].

Evaluating cumulative risk to environmental stressors is a key component of environmental justice. We explored interactions between melamine/AA concentrations and non-chemical stressors, a major strength of this study. Overall, we found little evidence of interaction, and for the evidence we did find, the direction of the effect was conflicting. A few combinations of chemical and non-chemical stressors showed increased odds of GDM per IQR increase in chemical concentration for those with higher levels of stress, while other combinations showed weaker associations between the chemicals and GDM among those with higher levels of non-chemical stress. However, this study was limited in its power to observe effects. Future, more highly powered studies are necessary to elucidate these trends.

Limited biomonitoring studies have assessed exposure to melamine and aromatic amines [14]. None of the chemicals included in the current study are measured in CDC’s NHANES (National Health and Nutrition Examination Survey), with the exception of melamine and cyanuric acid in the 2003–2004 survey [69]. Melamine concentrations in this study were comparable to exposure assessments in pregnant women in Taiwan [15, 70]. Melamine levels were also analogous to previous biomonitoring studies in the general US population [44, 71]. Cyanuric acid concentrations in this study (GM = 19.2 ng/mL) were higher than those reported by Zhu et al. who conducted a study in New York that used 213 repeated samples of 19 healthy participants in 2018 (GM = 9.26 ng/mL) [44] and the exposures reported in the 2003–2004 NHANES (GM = 5.86 ng/mL) [71]. Ammelide concentrations were also higher in our sample compared to the findings of Zhu and colleagues (GM = 4.0 vs 0.78 ng/mL) [44]. The median concentration of aniline in our sample (12.2 ng/mL) was higher than levels reported in a 2009 study of 1004 German volunteers (2.6 ng/mL), however, the median concentration of o-Anisidine was similar between studies (0.4 vs 0.3 ng/mL) [72].

There are several limitations of this study. First, we assessed a single urine sample for each participant, reflecting chemical concentration at a snapshot in time, which may not be an accurate representation of a participant’s exposure during the entire pregnancy. Our results may, therefore, be subject to bias from exposure misclassification as these chemicals have a short half-life in humans, ranging from hours to days [73, 74], though we expect this bias to be non-differential. Few studies have evaluated the intraindividual variability of melamine and AA levels over time [44]. Future research using repeat samples across pregnancy may be necessary to reduce measurement error. Second, the cross-sectional study design and timing of the exposure and outcome assessments are limitations to the study. Urine samples were collected during the second trimester, and gestational diabetes diagnosis is often conducted between 24 and 28 weeks’ gestation. For some participants, diagnosis of gestational diabetes may have occurred prior to urine sample collection. Future research using longitudinal study designs would be better suited to assess causal relationships between these exposures and outcomes. Third, the LOD divided by the square root of 2 may not precisely represent the distribution of aromatic amine levels below the limit of detection. Fourth, it is important to note that the association between the molar sum for each chemical class and GDM may largely be driven by a single chemical (for ΣMel, cyanuric acid, for ΣAA, aniline; see Fig. 1). Nonetheless, assessing combined exposure to multiple analytes is vital, as studies have found that melamine and its analogs may have synergistic effects [18]. Fifth, we were not able to investigate more subtle effects on blood glucose levels beyond a discrete GDM diagnosis due to limitations of our dataset. Sixth, many of the confidence intervals are wide, which may reflect a small sample size and low statistical power. As an observational study, there is also the possibility of residual confounding.

The findings from this study provide important insights into associations between chemical and psychosocial stressors and gestational diabetes but may not be generalizable to all pregnant women in the US due to the sociodemographic composition of our cohort. According to the CDC, between 2 and 10% of all pregnant women in the US are diagnosed with GDM annually [30]. The prevalence of GDM in this cohort (15%) is higher than in the general US population, which may be reflective of the high average age of participants in this cohort [33] and the proportion of participants identifying as Latinx or Asian, groups with high rates of GDM in the US [75]. The sample is highly educated and experiences little financial strain. Women with very high-risk pregnancies were also excluded from participation in the cohort, further limiting the external validity of the study. Additional studies are needed to assess how these relationships may differ across populations underrepresented in this cohort.

## Conclusion

Given rising rates of glucose abnormalities during pregnancy and our limited understanding of their etiology, identifying modifiable risk factors is critical in preventing GDM and its maternal and child health complications. In this study of 607 pregnant women in San Francisco, California, we found weak positive associations between melamine, o-Anisidine, and GDM. We also found evidence that prenatal perceived stress and discrimination are associated with GDM. We observed limited interaction between chemical and non-chemical stressors. Future studies should evaluate these stressors in other cohorts and continue to investigate the underlying biological mechanisms.

## Supplementary information

Supplementary information

## Data Availability

The sharing of anonymized data from this study is restricted due to ethical and legal restrictions. Data contains sensitive personal health information, which is protected under Health Insurance Portability and Accountability Act (HIPPA), thus making all data requests subject to Institutional Review Board (IRB) approval. Per University of California, San Francisco (UCSF) IRB, the data that support the findings of this study are restricted for transmission to those outside the primary investigative team. Data sharing with investigators outside the team requires IRB approval. Data requests may be submitted to the Program on Reproductive Health and the Environment (PRHE) by contacting Lynn Harvey at Lynn.Harvey@ucsf.edu. All requests for anonymized data will be reviewed by PRHE and then submitted to the UCSF IRB for approval.
