# Supplementary material for: Prenatal melamine, aromatic amine, and psychosocial stress exposures and their association with gestational diabetes mellitus in a San Francisco pregnancy cohort
Source: J Expo Sci Environ Epidemiol. 2025 Jun 30;35(6):907–20. doi: 10.1038/s41370-025-00787-x (PMC12583148; doi:10.1038/s41370-025-00787-x)
Supplement: Supplementary file 1 — Supplementary information [file 41370_2025_787_MOESM1_ESM.pdf]

Supplemental Material

Prenatal Melamine, Aromatic Amine, and Psychosocial Stress Exposures and Their Association with Gestational Diabetes Mellitus in a San Francisco Pregnancy Cohort

Emily Lasher,[1,2*] Jessica Trowbridge,[1] Alison Gemmill,[2] Rachel Morello-Frosch,[3] Erin DeMicco,[1] Kurunthachalam Kannan,[4] Jessie P. Buckley,[5] Tracey J. Woodruff[1]

[1]Department of Obstetrics, Gynecology and Reproductive Sciences, Program on Reproductive Health and the Environment, University of California, San Francisco, San Francisco, California, USA.
[2]Department of Population, Family, and Reproductive Health, Johns Hopkins Bloomberg School of Public Health, Baltimore, Maryland, USA.
[3]School of Public Health and Department of Environmental Science, Policy, and Management, University of California, Berkeley, California, USA.
[4]Wadsworth Center, New York State Department of Health, Empire State Plaza, Albany, New York, USA.
[5]Department of Epidemiology, Gillings School of Global Public Health, University of North Carolina at Chapel Hill, Chapel Hill, North Carolina, USA.

*Corresponding Author, Email: emily.lasher@ucsf.edu

Table of Contents

Table S1. Perceived Stress Scale (PSS-4)

Table S2. Center for Epidemiologic Studies Depression (CES-D-10) Scale

Table S3. List of aromatic amines analyzed in urine samples

Figure S1. Directed Acyclic Graphs (DAGs) used to identify potential confounding variables prior to analyses, informed by existing literature

Figure S2. Flow chart indicating participant exclusion from final cohort

Table S4. Characteristics of study participants between 2014 and 2021 in San Francisco, CA (stress models)

Table S5. Non-chemical stress levels among pregnant women in San Francisco by race/ethnicity

Table S6. Prevalence of GDM by race/ethnicity in our pregnancy cohort compared to national estimates

Table S7. Concentrations and distributions of urinary melamine, melamine analogs, and aromatic amines measured among pregnant women in San Francisco by race/ethnicity (ng/mL)

Figure S3. Distributions of urinary melamine, melamine analogs, and aromatic amines measured among pregnant women in San Francisco by stress (ng/mL)

Table S8. Adjusted and unadjusted odds ratios of the relationship between urinary chemical concentrations and gestational diabetes in total and sex-stratified models

Table S9. Adjusted and unadjusted odds ratios of the relationship between non-chemical stressors and gestational diabetes in total and sex-stratified models

Table S1. Perceived Stress Scale (PSS-4)

Questions	The next questions ask about your feelings and thoughts during the last five years. Your choices for answers are: never, almost never, sometimes, fairly often, or very often.<br><br>You were unable to control the important things in your life.<br>Confident about your ability to handle your personal problems.<br>Things were going your way.<br>Difficulties were piling up so high that you could not overcome them.
Scoring	Questions 1 and 4: 0 = Never; 1 = Almost Never; 2 = Sometimes; 3 = Fairly Often; 4 = Very Often<br>Questions 2 and 3: 4 = Never; 3 = Almost Never; 2 = Sometimes; 1 = Fairly Often; 0 = Very Often<br><br>Lowest score: 0<br>Highest score: 16<br><br>Higher scores are correlated with more stress.

Table S2. Center for Epidemiologic Studies Depression Scale (CES-D-10)

Questions

The following questions are about specific emotions you may have felt during the past year. Your answer choices are rarely, some of the time, often or most of the time.

During the past year, how often ...

1. Were you bothered by things that usually do not bother you?
2. Did you have trouble staying focused on what you were doing?
3. Did you feel depressed?
4. Did you feel that everything you did was an effort?
5. Did you feel hopeful about the future?
6. Did you feel fearful?
7. Was your sleep restless?
8. Were you happy?
9. Did you feel lonely?
10. Did you feel that you could get going?

Scoring

	Rarely	Some of the time	Often	Most of the time
Questions 5, 8 & 10	3	2	1	0
All other questions	0	1	2	3

The total score was calculated by finding the sum of the 10 items. We did not score the form if more than 2 items were missing. Any score equal to or above 10 is considered depressed.

Table S3. List of aromatic amines analyzed in urine samples

Full Chemical Group Name	Chemical Group Code	Full Analyte Name	Analyte Code	CAS #
Aromatic amines		4-aminoacetanilide	AA4	122-80-5
Aromatic amines	AA	2-aminobiphenyl	ABP2	90-41-5
Aromatic amines	AA	4-aminobiphenyl	ABP4	92-67-1
Aromatic amines	AA	3-(3-aminobenzyl)phenylamine	ABPA33	19471-12-6
Aromatic amines	AA	2-amino-6-methoxybenzothiazole	AMOBZT2	1747-60-0
Aromatic amines	AA	aniline	ANI	62-53-3
Aromatic amines	AA	benzidine	BD	92-87-5
Aromatic amines	AA	1,1-bis(4-aminophenyl)cyclohexane	BPCH11	3282-99-3
Aromatic amines	AA	3-chloroaniline	CA3	108-42-9
Aromatic amines	AA	4-chloroaniline	CA4	106-47-8
Aromatic amines	AA	4-chloro-o-toluidine	CTD4	95-69-2
Aromatic amines	AA	3,4-diaminoanisole + 2,4-diaminoanisole	DAAS34_DAAS24	102-51-2_615-05-4
Aromatic amines	AA	2,4-diaminotoluene	DAT24	95-80-7
Aromatic amines	AA	2,6-diaminotoluene	DAT26	823-40-5
Aromatic amines	AA	3,4-diaminotoluene	DAT34	496-72-0
Aromatic amines	AA	3,4-dichloroaniline	DCA34	95-76-1
Aromatic amines	AA	2,4-dimethylaniline	DMA24	95-68-1
Aromatic amines	AA	2,6-dimethylaniline	DMA26	87-62-7
Aromatic amines	AA	3,3'-dimethylbenzidine	DMBD33	119-93-7
Aromatic amines	AA	2,2'-dimethyl-(1,1'-biphenyl)4,4'-diamine	DMBPDA	84-67-3
Aromatic amines	AA	4-ethoxyaniline	EA4	156-43-4
Aromatic amines	AA	4,4'-methylenebis(2-chloroaniline)	MBCA44	101-14-4
Aromatic amines	AA	4,4'-methylenedianiline	MDA44	101-77-9
Aromatic amines	AA	4,4'-methylenedi-o-toluidine	MTD44	838-88-0
Aromatic amines	AA	2-naphthylamine	NA2	91-59-8
Aromatic amines	AA	ortho-anisidine	OANSD	90-04-0
Aromatic amines	AA	4,4'-oxydianiline	OD44	101-80-4
Aromatic amines	AA	o-dianisidine	ODAD	119-90-4
Aromatic amines	AA	ortho/meta-toluidine	OMTD	95-53-4_108-44-1
Aromatic amines	AA	para-anisidine	PANSD	104-94-9
Aromatic amines	AA	para-cresidine	PCD	120-71-8
Aromatic amines	AA	1,3-phenylenediamine	PLD13	108-45-2
Aromatic amines	AA	para-toluidine	PTD	106-49-0
Aromatic amines	AA	4,4'-thiodianiline	TD44	139-65-1
Aromatic amines	AA	2,4,5-trimethylaniline	TMA245	137-17-7
Aromatic amines	AA	2,4,6-trimethylaniline	TMA246	88-05-1

Figure S1. Directed Acyclic Graphs (DAGs) used to identify potential confounding variables prior to analyses, informed by existing literature

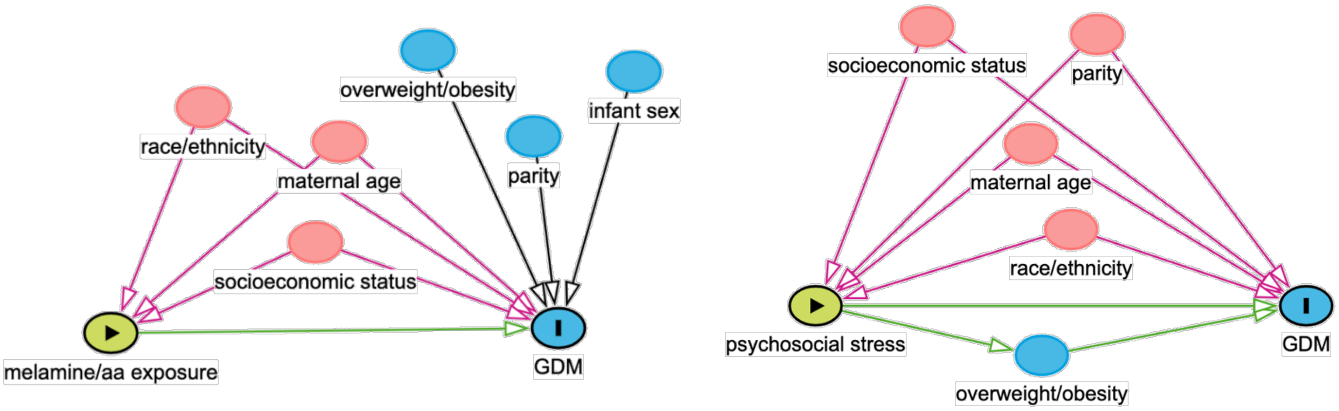

Green indicates exposure, blue indicates outcome, and covariates associated with outcome, and red indicates covariates associated with both exposure and outcome.

For a factor to be considered a potential confounder, it must be associated with the exposure and the disease and must not lie on the causal pathway. Though strongly associated with GDM, parity, BMI, and infant sex were not adjusted for in chemical models, as these covariates have not been found to be associated with exposure to melamine or aromatic amines.

Figure S2. Flow chart indicating participant exclusion from the final cohort

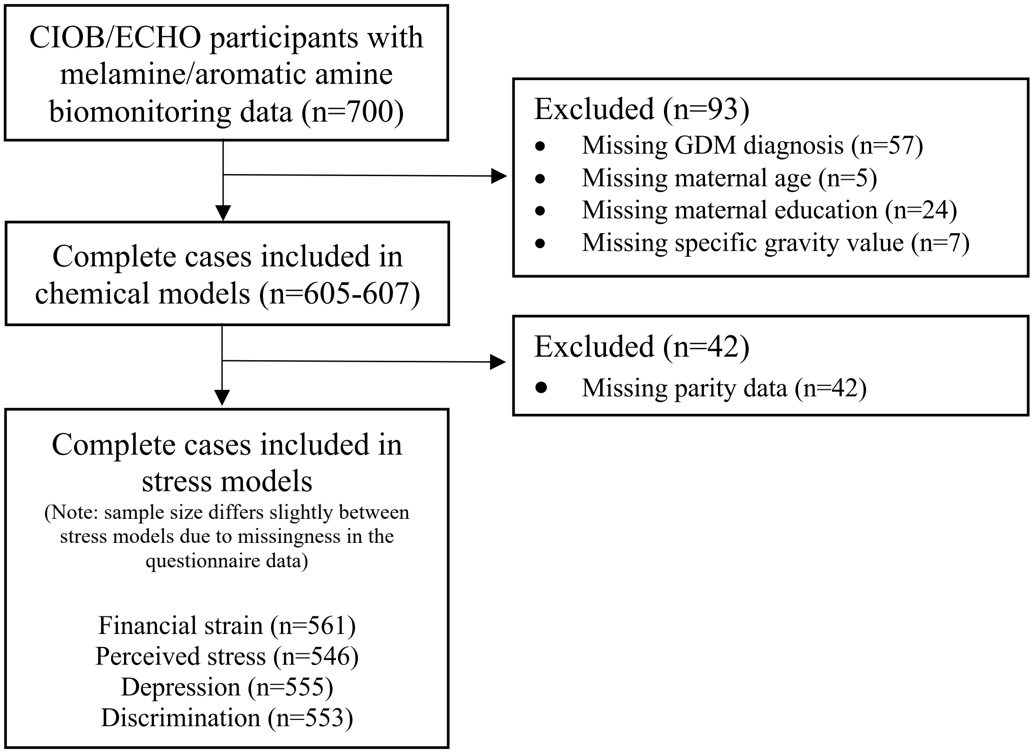

Table S4. Characteristics of study participants between 2014 and 2021 in San Francisco, CA *(stress models)*

	Mean ± SD or *n* (%)				
	Full sample (n=700)	Financial strain models (n=561)	Perceived stress models (n=546)	Depression models (n=555)	Discrimination models (n=553)
Covariates					
Maternal age at delivery (years)	33 ± 5.2	33 ± 4.9	33 ± 4.8	33 ± 4.8	33 ± 4.8
Race/ethnicity					
White	268 (38)	251 (45)	246 (45)	249 (45)	248 (45)
Latinx	245 (35)	148 (26)	141 (26)	146 (26)	147 (27)
Asian	118 (17)	109 (19)	108 (20)	108 (20)	107 (19)
Other or Unknown[a]	69 (10)	53 (10)	51 (9)	52 (9)	51 (9)
Education					
Some college or less	233 (35)	156 (28)	147 (27)	154 (28)	153 (28)
Bachelor's degree	168 (25)	156 (28)	154 (28)	154 (28)	154 (28)
Graduate degree	259 (39)	249 (44)	245 (45)	247 (45)	246 (44)
Missing	40	0	0	0	0
Pre-pregnancy BMI (kg/m^2)					
<18.5	15 (3)	14 (3)	14 (3)	14 (3)	14 (3)
18.5 - <25	286 (54)	262 (56)	253 (56)	260 (56)	256 (56)
25 - <30	130 (25)	112 (24)	111 (25)	112 (24)	114 (25)
≥30	95 (18)	77 (17)	74 (16)	77 (17)	76 (16)
Missing	174	96	94	92	93
Parity					
1+ births	324 (50)	270 (48)	261 (48)	266 (48)	267 (48)
No prior births	321 (50)	291 (52)	285 (52)	289 (52)	286 (52)
Missing	55	0	0	0	0
Infant sex assigned at birth					
Male	350 (51)	281 (50)	270 (50)	278 (50)	274 (50)
Female	341 (49)	279 (50)	275 (50)	276 (50)	278 (50)
Ambiguous or missing	9	1	1	1	1
Marital status					
Married/Living with Partner	566 (90)	505 (92)	492 (92)	499 (92)	496 (92)
Other	61 (10)	42 (8)	40 (8)	42 (8)	42 (8)
Missing	73	14	14	14	15
Measures of Non-Chemical Stress					
Financial strain					
Yes	236 (36)	163 (29)	153 (28)	162 (29)	160 (29)
No	422 (64)	398 (71)	390 (72)	391 (71)	390 (71)
Missing	42	0	3	2	3
Perceived stress (PSS-4)					
Low (0-3)	186 (29)	161 (30)	161 (30)	161 (30)	161 (30)
Moderate (4-6)	268 (42)	235 (43)	237 (43)	235 (43)	236 (43)
High (7+)	191 (30)	147 (27)	148 (27)	146 (27)	146 (27)
Missing	55	18	0	13	10
Depressive symptoms (CES-D-10)					
Low (0-4)	222 (34)	200 (36)	197 (36)	200 (36)	200 (36)
Moderate (5-9)	276 (42)	235 (43)	234 (43)	235 (42)	234 (43)
High (>10)	154 (24)	118 (21)	111 (21)	120 (22)	115 (21)

Missing	48	8	4	0	4
Discrimination[b]					
Never	225 (34)	196 (36)	194 (36)	195 (36)	197 (36)
Rarely	253 (39)	223 (40)	219 (40)	223 (40)	223 (40)
Sometimes/often/very often	178 (27)	131 (24)	130 (24)	131 (24)	133 (24)
Missing	44	11	3	6	0
Outcome					
GDM					
No	552 (86)	476 (85)	461 (84)	469 (85)	467 (84)
Yes	91 (14)	85 (15)	85 (16)	86 (15)	86 (16)
Missing	57	0	0	0	0

SD = standard deviation, GDM = gestational diabetes mellitus, BMI = body mass index (calculated as kilograms per meter squared, categories are based on CDC guidelines), PSS-4 = Perceived Stress Scale, CES-D-10 = Center for Epidemiologic Studies Depression Scale

[a]Race and ethnicity categories were collapsed for use in logistic regression models due to small numbers in some categories. Other/unknown includes Black or African American, Native Hawaiian or Pacific Islander, Native American, multiracial, and individuals of unknown race.

[b]Response to the question, "How often do you feel that you, personally, have been discriminated against because of your race, ethnicity, ancestry, religion, or color?"

Table S5. Non-chemical stress levels among pregnant women in San Francisco by race/ethnicity

Measures of Non-Chemical Stress	*n* (%)			
	White (n=255)	Latinx (n=184)	Asian (n=111)	Other/Unknown (n=57)
Financial strain				
Yes	17 (7)	126 (73)	18 (16)	26 (47)
No	238 (93)	47 (27)	92 (84)	29 (53)
Missing	0	11	1	2
Perceived Stress (PSS-4)				
Low (0-3)	89 (36)	43 (26)	34 (31)	8 (15)
Moderate (4-6)	111 (44)	51 (31)	54 (50)	28 (52)
High (7+)	50 (20)	71 (43)	21 (19)	18 (33)
Missing	5	19	2	3
Depression (CES-D-10)				
Low (0-4)	115 (46)	45 (26)	35 (32)	10 (19)
Moderate (5-9)	102 (40)	69 (41)	54 (50)	26 (48)
High (>10)	36 (14)	56 (33)	20 (18)	18 (33)
Missing	2	14	2	3
Discrimination				
Never	128 (51)	51 (29)	15 (14)	13 (24)
Rarely	109 (43)	54 (31)	49 (45)	21 (39)
Sometimes/often/very often	15 (6)	69 (40)	44 (41)	20 (37)
Missing	3	10	3	3

Table S6. Prevalence of GDM by race/ethnicity in our pregnancy cohort compared to national estimates

	CIOB/ECHO Cohort (2014-2021)	United States (2016)[a]
White	12.2	5.3
Latinx	15.8	6.6
Asian	17.1	11.1

[a]Deputy NP, Kim SY, Conrey EJ, Bullard KM. Prevalence and changes in preexisting diabetes and gestational diabetes among women who had a live birth: United States, 2012-2016. *MMWR Morb Mortal Wkly Rep*. 2018;67(43):1201-1207. doi:10.15585/mmwr.mm6743a2

Table S7. Concentrations and distributions of urinary melamine and its analogs, and aromatic amines detected in ≥20% of samples from pregnant women in San Francisco by race/ethnicity (ng/mL)

Chemical		n	LOD	Race/Ethnicity	% Above LOD (n)[a]	*SG-adjusted concentrations*					
						GM (GSD)[b]	Min	Percentile			Max
								25	50	75	
Melamine & its analogs											
Melamine	MEL	607	0.090	White	100 (255)	2.0 (3.5)	<LOD	0.9	1.7	3.3	171
				Latinx	100 (184)	1.7 (2.8)	0.3	0.8	1.4	3.0	72
				Asian	100 (111)	2.0 (4.1)	0.2	0.8	1.7	3.6	4186
				Other/Unknown	100 (57)	2.0 (2.7)	0.4	1.1	1.8	3.4	56
Cyanuric Acid	CYA	607	0.156	White	100 (255)	21.2 (2.9)	0.7	12.4	18.3	28.8	16240
				Latinx	100 (184)	16.6 (1.9)	4.7	10.8	15.6	22.5	102
				Asian	100 (111)	19.1 (2.9)	1.0	9.7	16.6	29.3	4340
				Other/Unknown	100 (57)	19.6 (2.1)	6.9	12.2	17.8	24.8	382
Ammelide	AMD	607	0.135	White	99 (254)	4.6 (2.8)	0.3	2.6	4.4	7.2	325
				Latinx	99 (182)	3.4 (2.2)	<LOD	2.2	3.5	5.2	31
				Asian	99 (110)	3.6 (2.6)	<LOD	2.3	3.5	5.7	295
				Other/Unknown	100 (57)	4.2 (2.5)	0.3	2.4	4.1	6.2	53
ΣMelamine[c]	ΣMEL	607	NA	White	NA	0.2 (2.8)	<0.1	0.1	0.2	0.3	126
				Latinx		0.2 (1.8)	0.1	0.1	0.2	0.3	1.0
				Asian		0.2 (2.8)	<0.1	0.1	0.2	0.3	69
				Other/Unknown		0.2 (2.0)	0.1	0.1	0.2	0.3	3.5
Aromatic amines											
Aniline	ANI	605	0.100	White	95 (241)	14.4 (5.6)	<LOD	9.0	14.1	25.0	102760
				Latinx	99 (183)	12.2 (2.4)	0.1	7.1	11.9	19.9	172
				Asian	94 (104)	9.3 (3.6)	0.1	5.3	10.1	19.1	449
				Other/Unknown	95 (54)	9.6 (3.4)	<LOD	8.2	10.2	15.9	113
4-Chloroaniline	CA4	605	0.050	White	83 (211)	0.7 (5.1)	<LOD	0.3	0.7	1.7	174
				Latinx	91 (167)	0.9 (3.7)	<LOD	0.5	0.9	1.8	50
				Asian	81 (90)	0.6 (4.9)	<LOD	0.2	0.6	1.6	116
				Other/Unknown	86 (49)	0.9 (5.1)	<LOD	0.4	0.8	1.6	169
4,4′-Methylenedianiline	MDA44	605	0.030	White	84 (213)	0.9 (7.8)	<LOD	0.4	1.3	2.2	630
				Latinx	87 (160)	0.8 (6.3)	<LOD	0.5	1.1	2.0	552
				Asian	77 (85)	0.7 (9.7)	<LOD	0.2	1.0	2.5	549
				Other/Unknown	82 (47)	0.9 (7.1)	<LOD	0.5	1.1	2.2	59
o-Anisidine	OANSD	605	0.030	White	71 (182)	0.5 (7.7)	<LOD	0.1	0.8	1.9	72
				Latinx	64 (118)	0.3 (7.3)	<LOD	<0.1	0.4	1.1	43
				Asian	69 (77)	0.4 (6.1)	<LOD	0.1	0.5	1.5	30
				Other/Unknown	67 (38)	0.3 (7.2)	<LOD	<0.1	0.5	1.0	8.5
Composite of m/o-Toluidine	OMTD	605	0.050	White	85 (217)	1.5 (5.6)	<LOD	0.7	1.7	3.4	252
				Latinx	95 (174)	1.5 (3.0)	<LOD	0.9	1.4	3.0	29
				Asian	83 (92)	1.1 (4.3)	<LOD	0.7	1.2	2.8	89
				Other/Unknown	84 (48)	1.1 (4.3)	<LOD	0.8	1.4	2.4	15
3-Chloroaniline	CA3	605	0.050	White	37 (95)	NA	<LOD	<LOD	0.1	1.0	353
				Latinx	39 (72)		<LOD	<LOD	0.1	0.9	278
				Asian	46 (51)		<LOD	<LOD	0.1	0.8	1778

				Other/Unknown	47 (27)		<LOD	<LOD	0.2	1.2	19
3,4-Dichloroaniline	DCA34	605	0.100	White	31 (78)	NA	<LOD	<LOD	0.1	2.9	173
				Latinx	30 (55)		<LOD	<LOD	<LOD	2.3	67
				Asian	35 (39)		<LOD	<LOD	0.2	3.4	102
				Other/Unknown	25 (14)		<LOD	<LOD	0.1	1.0	12
p-Toluidine	PTD	605	0.030	White	26 (66)	NA	<LOD	<LOD	<0.1	0.8	8.0
				Latinx	48 (88)		<LOD	<LOD	0.1	1.6	21
				Asian	22 (24)		<LOD	<LOD	<0.1	0.1	29
				Other/Unknown	40 (23)		<LOD	<LOD	<0.1	1.2	5.5
ΣAA[c]	ΣAA	605	NA	White	NA	0.3 (4.1)	<0.1	0.2	0.2	0.5	1106
				Latinx		0.2 (2.1)	<0.1	0.2	0.2	0.4	3.4
				Asian		0.2 (3.1)	<0.1	0.1	0.2	0.3	22
				Other/Unknown		0.2 (2.2)	<0.1	0.2	0.2	0.3	1.3

LOD = limit of detection, GM = geometric mean, GSD = geometric standard deviation, Min = minimum, Max = maximum, SG = specific gravity

[a]Detection frequency was calculated from unadjusted values.

[b]Calculated for analytes with ≥65% above the LOD.

[c]Molar sums were calculated by summing the concentrations of frequently detected analytes (detection frequency >20%) scaled by their molecular weight. The units for these values are nmol/mL.

Figure S3. Distributions of urinary melamine, melamine analogs, and aromatic amines measured among pregnant women in San Francisco by stress (ng/mL)

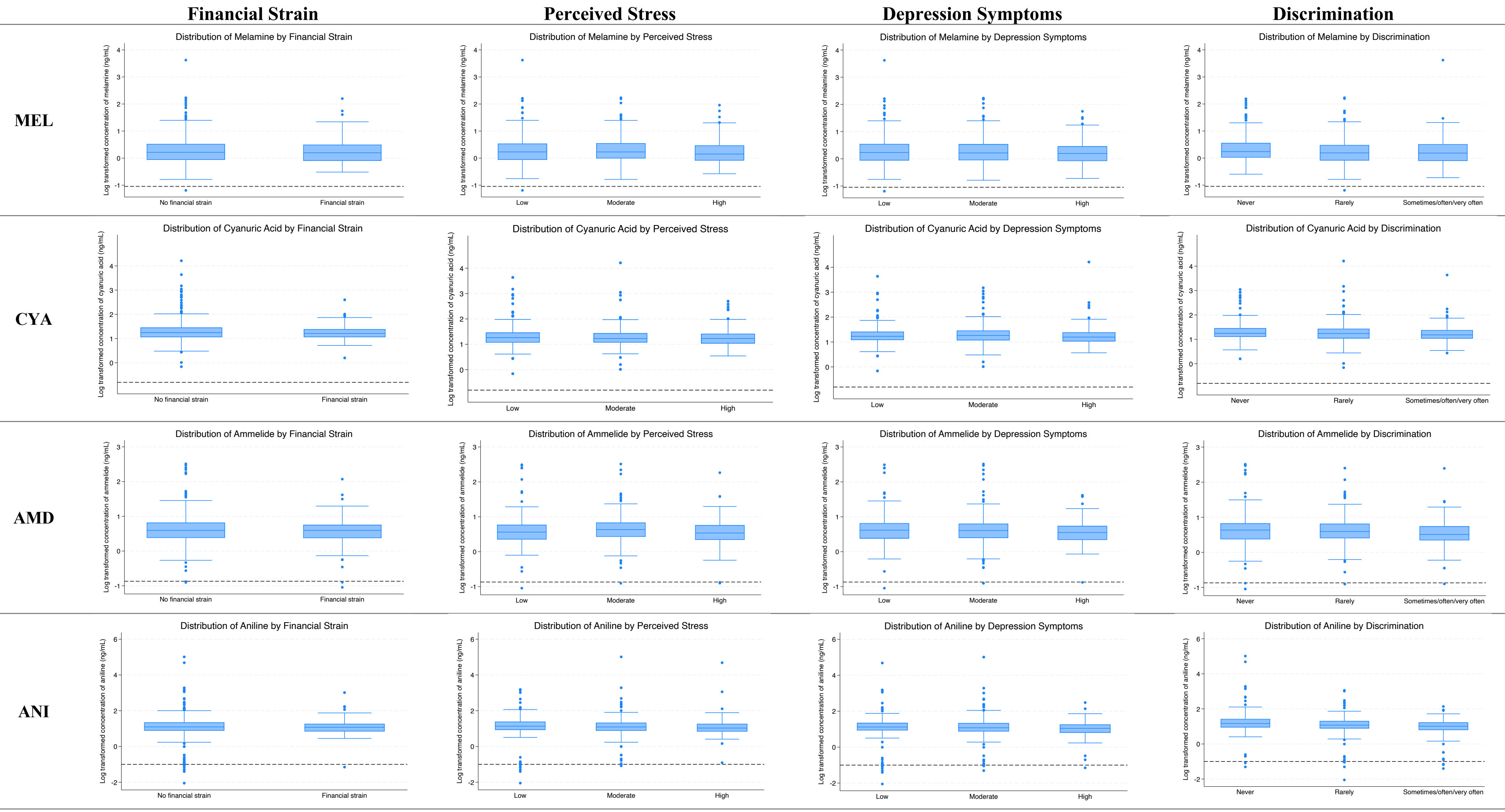

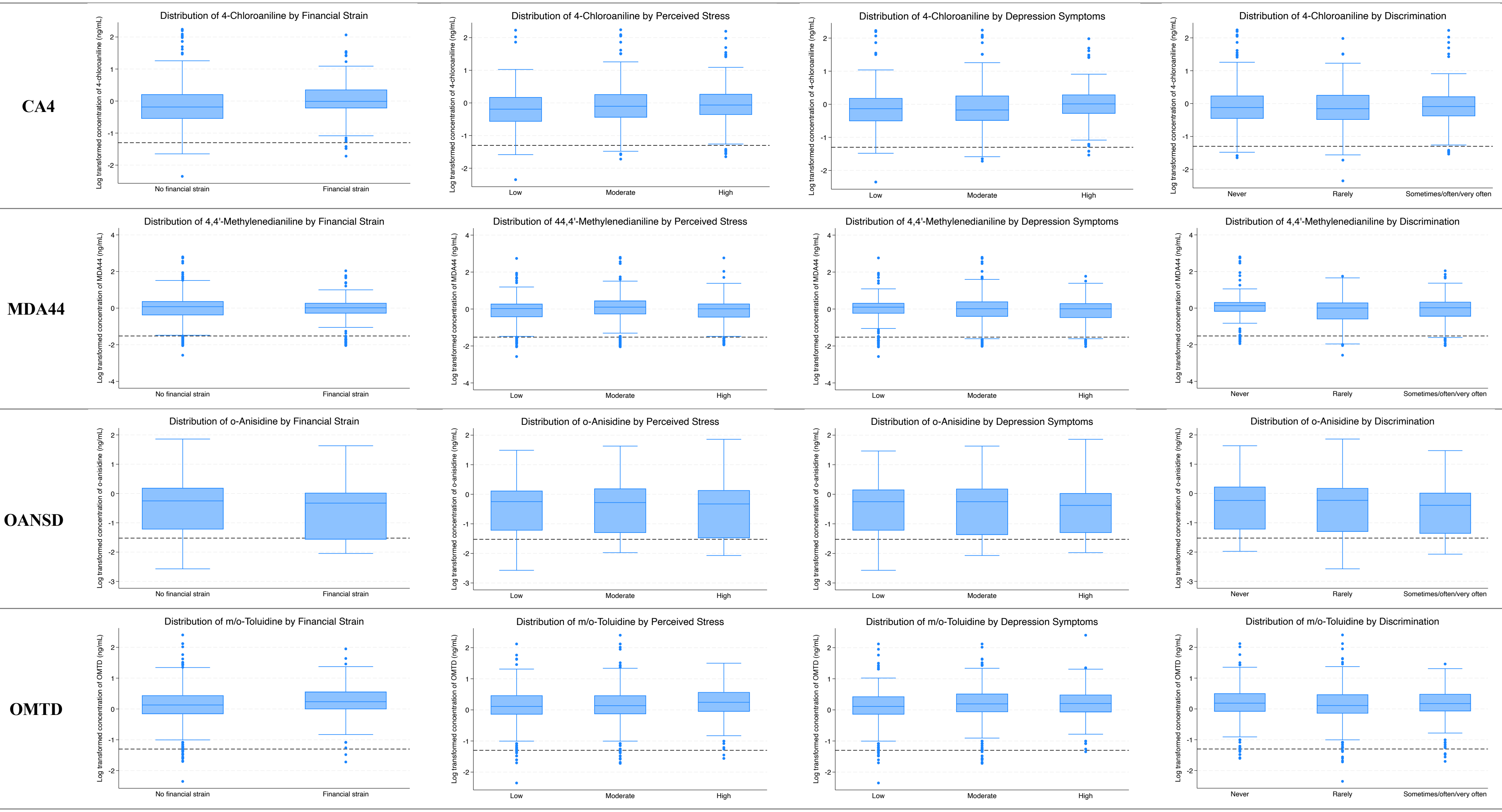

Note: Dashed line denotes the limit of detection.

Table S8. Adjusted and unadjusted odds ratios of the relationship between urinary chemical concentrations and gestational diabetes in total and sex-stratified models

Exposure	Model		Odds Ratio (95% CI)
Melamine & Melamine Analogs (n=607)			
ΣMelamine (IQR: 0.2)	Total	Unadjusted	0.99 (0.96 – 1.03)
		Adjusted[a]	0.99 (0.96 – 1.03)
	Male[b]	Unadjusted	1.05 (0.98 – 1.14)
		Adjusted	1.08 (1.00 – 1.17)
	Female	Unadjusted	0.96 (0.88 – 1.05)
		Adjusted	0.96 (0.87 – 1.05)
Melamine (IQR: 2.4)	Total	Unadjusted	0.98 (0.93 – 1.03)
		Adjusted	0.98 (0.94 – 1.03)
	Male	Unadjusted	1.02 (0.95 – 1.09)
		Adjusted	1.03 (0.97 – 1.11)
	Female	Unadjusted	0.94 (0.84 – 1.05)
		Adjusted	0.94 (0.83 – 1.06)
Cyanuric Acid (IQR: 15)	Total	Unadjusted	0.99 (0.96 – 1.02)
		Adjusted	0.99 (0.96 – 1.02)
	Male	Unadjusted	1.05 (0.98 – 1.12)
		Adjusted	1.07 (0.99 – 1.14)
	Female	Unadjusted	0.97 (0.91 – 1.04)
		Adjusted	0.97 (0.90 – 1.04)
Ammelide (IQR: 3.8)	Total	Unadjusted	1.00 (0.98 – 1.03)
		Adjusted	1.01 (0.98 – 1.04)
	Male	Unadjusted	1.04 (0.99 – 1.09)
		Adjusted	1.05 (0.99 – 1.12)
	Female	Unadjusted	0.99 (0.94 – 1.03)
		Adjusted	0.98 (0.93 – 1.04)
Aromatic Amines (n=605)			
ΣAA (IQR: 0.2)	Total	Unadjusted	1.00 (0.99 – 1.01)
		Adjusted	1.00 (0.99 – 1.01)
	Male	Unadjusted	1.01 (0.99 – 1.03)
		Adjusted	1.02 (0.99 – 1.04)
	Female	Unadjusted	0.98 (0.91 – 1.05)
		Adjusted	0.97 (0.90 – 1.05)
Aniline (IQR: 13.7)	Total	Unadjusted	0.99 (0.96 – 1.02)
		Adjusted	0.99 (0.95 – 1.03)
	Male	Unadjusted	0.99 (0.95 – 1.04)
		Adjusted	1.00 (0.96 – 1.05)
	Female	Unadjusted	0.98 (0.93 – 1.04)
		Adjusted	0.98 (0.92 – 1.05)
4-Chloroaniline (IQR: 1.3)	Total	Unadjusted	0.92 (0.82 – 1.04)
		Adjusted	0.91 (0.80 – 1.04)
	Male	Unadjusted	0.84 (0.62 – 1.13)
		Adjusted	0.77 (0.54 – 1.09)
	Female	Unadjusted	0.95 (0.85 – 1.06)
		Adjusted	0.96 (0.88 – 1.04)
4,4′-Methylenedianiline (IQR: 1.7)	Total	Unadjusted	1.00 (0.99 – 1.01)

		Adjusted	1.00 (0.99 – 1.01)
	Male	Unadjusted	1.00 (0.99 – 1.01)
		Adjusted	1.01 (1.00 – 1.01)
	Female	Unadjusted	0.99 (0.97 – 1.02)
		Adjusted	0.99 (0.96 – 1.02)
o-Anisidine (IQR: 1.3)	Total	Unadjusted	1.04 (0.99 – 1.08)
		Adjusted	1.03 (0.98 – 1.08)
	Male	Unadjusted	1.18 (1.04 – 1.33)*
		Adjusted	1.18 (1.03 – 1.36)*
	Female	Unadjusted	1.00 (0.93 – 1.07)
		Adjusted	0.99 (0.92 – 1.06)
Composite of o/m-Toluidine (IQR: 2.2)	Total	Unadjusted	0.99 (0.94 – 1.04)
		Adjusted	0.99 (0.94 – 1.04)
	Male	Unadjusted	0.99 (0.90 – 1.10)
		Adjusted	0.99 (0.90 – 1.10)
	Female	Unadjusted	0.99 (0.93 – 1.04)
		Adjusted	0.99 (0.93 – 1.04)
3-Chloroaniline (binary)	Total	Unadjusted	1.09 (0.70 – 1.72)
		Adjusted	1.11 (0.70 – 1.76)
	Male	Unadjusted	0.96 (0.51 – 1.81)
		Adjusted	0.90 (0.47 – 1.75)
	Female	Unadjusted	1.25 (0.65 – 2.41)
		Adjusted	1.47 (0.74 – 2.92)
3,4-Dichloroaniline (binary)	Total	Unadjusted	0.48 (0.27 – 0.84)*
		Adjusted	0.47 (0.27 – 0.83)*
	Male	Unadjusted	0.39 (0.17 – 0.90)*
		Adjusted	0.33 (0.14 – 0.79)*
	Female	Unadjusted	0.59 (0.28 – 1.24)
		Adjusted	0.60 (0.28 – 1.31)
p-Toluidine (binary)	Total	Unadjusted	1.01 (0.63 – 1.63)
		Adjusted	1.03 (0.63 – 1.71)
	Male	Unadjusted	1.45 (0.77 – 2.73)
		Adjusted	1.18 (0.59 – 2.35)
	Female	Unadjusted	0.65 (0.31 – 1.38)
		Adjusted	0.76 (0.35 – 1.67)

[a]Adjusted model controls for maternal age, race/ethnicity, and education.
[b]Two participants with missing infant sex were excluded from sex-stratified models.
* $p<0.05$

Table S9. Adjusted and unadjusted odds ratios of the relationship between non-chemical stressors and gestational diabetes in total and sex-stratified models

Exposure	Model		N	Odds Ratio (95% CI)[a]		N	Odds Ratio (95% CI)[b]
Financial Strain	Total	Unadjusted	561	No	ref	537	ref
				Yes	1.02 (0.61 – 1.69)		1.03 (0.61 – 1.72)
		Adjusted[c]		No	ref		ref
				Yes	0.71 (0.34 – 1.50)		0.63 (0.29 – 1.36)
	Male[d]	Unadjusted	281	No	ref	265	ref
				Yes	1.37 (0.69 – 2.73)		1.39 (0.68 – 2.82)
		Adjusted		No	ref		ref
				Yes	0.61 (0.21 – 1.76)		0.53 (0.18 – 1.60)
	Female	Unadjusted	279	No	ref	271	ref
				Yes	0.73 (0.34 – 1.57)		0.74 (0.35 – 1.60)
		Adjusted		No	ref		ref
				Yes	0.74 (0.25 – 2.16)		0.67 (0.23 – 2.01)
Perceived Stress (PSS-4)	Total	Unadjusted	546	Low (0-3)	ref	537	ref
				Moderate (4-6)	1.06 (0.59 - 1.89)		1.08 (0.61 – 1.92)
				High (7+)	1.54 (0.84 – 2.82)		1.54 (0.84 – 2.83)
		Adjusted		Low (0-3)	ref		ref
				Moderate (4-6)	1.02 (0.57 – 1.84)		1.06 (0.59 – 1.91)
				High (7+)	1.41 (0.73 – 2.70)		1.40 (0.73 – 2.69)
	Male	Unadjusted	270	Low (0-3)	ref	265	ref
				Moderate (4-6)	1.15 (0.48 – 2.75)		1.16 (0.49 – 2.78)
				High (7+)	2.34 (0.97 – 5.65)		2.32 (0.95 – 5.68)
		Adjusted		Low (0-3)	ref		ref
				Moderate (4-6)	1.18 (0.48 – 2.87)		1.19 (0.49 – 2.91)
				High (7+)	1.80 (0.70 – 4.59)		1.81 (0.71 – 4.66)
	Female	Unadjusted	275	Low (0-3)	ref	271	ref
				Moderate (4-6)	1.00 (0.46 – 2.18)		1.03 (0.48 – 2.25)
				High (7+)	1.04 (0.44 – 2.45)		1.06 (0.45 – 2.49)
		Adjusted		Low (0-3)	ref		ref
				Moderate (4-6)	0.92 (0.41 – 2.06)		0.96 (0.43 – 2.15)
				High (7+)	1.16 (0.46 – 2.97)		1.16 (0.45 – 2.96)
Depression (CES-D-10)	Total	Unadjusted	555	Low (0-4)	ref	537	ref
				Moderate (5-9)	1.06 (0.63 – 1.79)		1.05 (0.62 – 1.77)
				High (>10)	1.07 (0.57 – 1.99)		1.05 (0.55 – 2.01)
		Adjusted		Low (0-4)	ref		ref
				Moderate (5-9)	0.94 (0.55 – 1.63)		0.94 (0.55 – 1.62)
				High (>10)	0.86 (0.44 – 1.66)		0.86 (0.44 – 1.70)
	Male	Unadjusted	278	Low (0-4)	ref	265	ref
				Moderate (5-9)	1.40 (0.66 – 2.99)		1.42 (0.67 – 3.03)
				High (>10)	1.41 (0.60 – 3.32)		1.34 (0.54 – 3.33)
		Adjusted		Low (0-4)	ref		ref
				Moderate (5-9)	1.25 (0.57 – 2.75)		1.30 (0.59 – 2.84)
				High (>10)	1.06 (0.42 – 2.63)		1.04 (0.40 – 2.70)
	Female	Unadjusted	276	Low (0-4)	ref	271	Ref
				Moderate (5-9)	0.81 (0.39 – 1.67)		0.79 (0.38 – 1.63)
				High (>10)	0.78 (0.31 – 1.97)		0.81 (0.32 – 2.05)
		Adjusted		Low (0-4)	ref		ref
				Moderate (5-9)	0.71 (0.33 – 1.54)		0.70 (0.32 – 1.51)
				High (>10)	0.70 (0.26 – 1.90)		0.72 (0.26 – 1.95)
Discrimination	Total	Unadjusted	553	never	ref	537	ref

			rarely	1.99 (1.11 – 3.57)*		1.91 (1.06 – 3.44)*
			sometimes/often/ very often	2.50 (1.33 – 4.69)*		2.48 (1.31 – 4.69)*
	Adjusted		never	ref		ref
			rarely	2.12 (1.15 – 3.91)*		2.08 (1.12 – 3.85)*
			sometimes/often/ very often	2.33 (1.16 – 4.67)*		2.38 (1.18 – 4.81)*
Male	Unadjusted	274	never	ref	265	ref
			rarely	1.80 (0.78 – 4.15)		1.66 (0.71 – 3.86)
			sometimes/often/ very often	2.57 (1.09 – 6.06)*		2.46 (1.03 – 5.88)*
	Adjusted		never	ref		ref
			rarely	1.76 (0.72 – 4.31)		1.69 (0.69 – 4.18)
			sometimes/often/ very often	1.98 (0.75 – 5.24)		2.01 (0.75 – 5.38)
Female	Unadjusted	278	never	ref	271	ref
			rarely	2.15 (0.94 – 4.91)		2.14 (0.94 – 4.90)
			sometimes/often/ very often	2.37 (0.93 – 6.01)		2.46 (0.97 – 6.28)
	Adjusted		never	ref		ref
			rarely	2.56 (1.07 – 6.11)*		2.58 (1.08 – 6.18)*
			sometimes/often/ very often	2.72 (0.95 – 7.76)		2.99 (1.04 – 8.59)*

[a]Sample size differs between exposure groups due to missingness in the data. See Table S4 for additional information.
[b]Models are restricted to the same analytic population.
[c]Adjusted models control for maternal age, race/ethnicity, education, and parity.
[d]One participant with missing infant sex was excluded from sex-stratified models.
* $p<0.05$
